# Isolation and Characterization of an Adult Stem Cell Population from Human Epidural Fat

**DOI:** 10.1155/2019/2175273

**Published:** 2019-03-17

**Authors:** Nedaa Al-Jezani, Roger Cho, Anand O. Masson, Brian Lenehan, Roman Krawetz, Frank G. Lyons

**Affiliations:** ^1^McCaig Institute for Bone and Joint Health, University of Calgary, Canada; ^2^Medical Science Graduate Program, University of Calgary, Canada; ^3^Division of Orthopaedic Surgery, Foothills Medical Centre, Calgary, Canada; ^4^Biomedical Engineering Graduate Program, University of Calgary, Canada; ^5^University Hospital Limerick and Mater Misericordiae University Hospital, Ireland

## Abstract

**Study Design:**

Isolation and characterization of human epidural fat (HEF) stem/progenitor cells.

**Objective:**

To identify a progenitor population within HEF and to determine if they meet the minimal criteria of a mesenchymal stem cell (MSC).

**Summary of Background Data:**

The biological function, if any, has yet to be determined for HEF. The presence of MSCs within HEF may indicate a regenerative potential within the HEF.

**Methods:**

HEF was isolated from 10 patients during elective spinal surgery. HEF cells were differentiated along osteo-, adipo-, and chondrogenic lineages, with differentiation analyzed via qPCR and histology. The cell surface receptor profile of HEF cells was examined by flow cytometry. HEF cells were also assayed through the collagen contraction assay. *Prx1*^CreERT2GFP^:*R26R*^TdTomato^ MSC lineage-tracking mice were employed to identify EF MSCs *in vivo*.

**Results:**

HEF cell lines were obtained from all 10 patients in the study. Cells from 2/10 patients demonstrated full MSC potential, while cells from 6/10 patients demonstrated progenitor potential; 2/10 patients presented with cells that retained only adipogenic potential. HEF cells demonstrated MSC surface marker expression. All patient cell lines contracted collagen gels. A *Prx1*-positive population in mouse epidural fat that appeared to contribute to the dura of the spinal cord was observed *in vivo*.

**Conclusions:**

MSC and progenitor populations are present within HEF. MSCs were not identified in all patients examined in the current study. Furthermore, all patient lines demonstrated collagen contraction capacity, suggesting either a contaminating activated fibroblast population or HEF MSCs/progenitors also demonstrating a fibroblast-like phenotype. *In vivo* analysis suggests that these cell populations may contribute to the dura. Overall, these results suggest that cells within epidural fat may play a biological role within the local environment above providing a mechanical buffer.

## 1. Introduction

Mesenchymal stem cells (MSCs) are well known for their self-renewal capacity and ability to differentiate into multiple cell lineages [1]. There is also evidence demonstrating that MSCs can direct repair through immune-modulating properties [2–6]. Specifically, MSCs can regulate the proliferation, activity, and differentiation of lymphocytes [7, 8] and natural killer cells [9]. For these attractive properties, MSCs have been employed in clinical trials for numerous disorders [10–13], yet it could be argued that we still lack a clear understanding of their roles within adult tissues.

MSCs were first described by Friedenstein et al. in bone marrow [14] and subsequently isolated by Pittinger [1]. MSCs have also been successfully isolated from synovium [15], umbilical cord blood [16], lung [17], muscles [18], adipose tissue [19], dental pulp [20], pancreas [21], and others. The most common sources of MSCs in preclinical and clinical trials are bone marrow [22–25] and adipose tissue [26–28]. Most anatomical sites of fat in the human body have been characterized, including their potential as a source of MSCs [19, 29, 30]; however, little is known about the role of human epidural fat (HEF). Routinely during spinal surgery, epidural fat is debrided and discarded; however, studies of HEF are extremely limited and a biological characterization of HEF has never been undertaken to our knowledge. In 1997, Beaujeux et al. concluded that HEF was a functional tissue (based on histological analysis) [31]. In 2009, an electron microscopy study proposed that HEF served as a mechanical buffer to provide cushion or support to the thecal sac within the spinal canal [32], yet this has not been demonstrated at the functional level. The knee fat pad is an anatomical fat site also once considered to serve solely as a mechanical buffer, but this has been studied extensively in recent years [30, 33, 34]. The knee fat pad is now considered biologically active and contains MSCs that can be induced to differentiate into multiple cell lineages [30, 35–38], with similar results observed in other fat types [19, 39]. Therefore, rather than being biologically redundant or a supposed mechanical buffer, the fat pad may promote some level of tissue maintenance and repair in the synovial environment but, at the least, contains cells that have regenerative potential *in vitro* [30, 36]. These and other cellular studies on fat sites within the body serve as a rationale to investigate the biological characteristics of HEF and to examine it as a potential source of MSC and/or progenitor cell populations. If it is, this may force a reexamination of our current thinking of epidural fat, and whether or not we should continue to debride and discard it routinely.

## 2. Materials and Methods

### 2.1. Ethics Statement and Demographic Characteristics

Institutional ethical approval was granted by the University of Calgary Research Ethics Board (ID: REB17-0220). All patients signed informed consent forms for the release of HEF, debrided during the routine course of the surgical procedure. Age and gender were recorded for each patient. This study was carried out in accordance with the declaration of Helsinki.

HEF from the lumbar spinal canal was isolated from 10 patients (5 M/5 F; 24-80 years old) during primary elective posterior spinal decompression for degenerative lumbar spinal stenosis. All patients had an ASA score of 1 or 2, and patients with a history of malignancy, autoimmune disease, or inflammatory arthropathy were excluded.

### 2.2. Epidural Fat Digestion

HEF samples were digested at 37°C for 90 minutes with filtered 1 mg/mL type IV collagenase (Sigma-Aldrich, St. Louis, Missouri). The cell suspension was filtered at 70 *μ*m and washed twice with DPBS (Lonza BioWhittaker, Walkersville, Maryland), then seeded in MSC media (details below) in 12-well plates and incubated at 37°C and 5% CO_2_. At 70% confluency, cells were washed with DPBS and passaged with trypsin (Lonza BioWhittaker). The cells were allowed to proliferate with medium changes performed every 2 days.

### 2.3. Differentiation Analysis

The expanded cells were induced to differentiate into bone, cartilage, and fat. The detailed protocols are presented as follows:

#### 2.3.1. Osteogenic Differentiation

Cells were seeded as monolayers (5 × 10^5^ cells/well in 24-well plates) in DMEM/F-12 supplemented with 10% FBS, 1% antibiotic-antimycotic (A.A.), 1% MEM nonessential amino acids (NEAA) (all from Thermo Fisher Scientific), and osteogenesis-inducing agents including dexamethasone (Dex 10^−4^ M), L-ascorbic acid (50 *μ*g/mL), and *β*-glycerophosphate (10 mM) (all from Sigma-Aldrich). This differentiation protocol was undertaken for 21 days with medium changes every two days.

#### 2.3.2. Chondrogenic Differentiation

Cells were pelleted (5 × 10^5^ cells per pellet, centrifuged at 5000 rpm for 6 minutes), and chondrogenesis was induced by culturing the pellets in DMEM/F-12 supplemented with 1% A.A., 1% NEAA (all from Thermo Fisher Scientific), and chondrogenesis-inducing agents including dexamethasone (10 nM) (Sigma-Aldrich), ascorbic acid (50 *μ*g/mL) (Sigma-Aldrich), TGF-*β*3 (10 ng/mL) (PeproTech Inc., Rocky Hill, New Jersey), BMP-2 (500 ng/mL) (PeproTech Inc.), sodium pyruvate (Thermo Fisher Scientific), and insulin transferrin selenium (ITS) (Lonza BioWhittaker). This differentiation protocol was undertaken for 21 days with medium changes every two days.

#### 2.3.3. Adipogenic Differentiation

Cells were seeded as monolayers (5 × 10^5^ cells/well in 24-well plates) in DMEM/F-12 supplemented with 10% FBS, 1% A.A., 1% NEAA (all from Thermo Fisher Scientific), and adipogenesis-inducing agents including dexamethasone (1 *μ*M), insulin (10 *μ*M), indomethacin (200 *μ*M), and isobutylmethylxanthine (500 *μ*M) (all from Sigma-Aldrich). Medium changes were performed twice a week during the 21-day differentiation protocol.

### 2.4. Quantitative PCR (qPCR)

To collect mRNA, two different methods were used depending on the differentiation procedure. mRNA from cells that underwent osteogenic and adipogenic differentiation was isolated via a TRIzol reagent (Thermo Fisher Scientific), while the mRNA from cells that underwent chondrogenic differentiation was extracted by a Total RNA Kit I (Omega Bio-tek, VWR). Common elements to both protocols include the addition of 1 mL of the TRIzol reagent with glycogen (Thermo Fisher Scientific) to the cells, followed by the addition of 200 *μ*L of chloroform (Sigma-Aldrich). The samples were vortexed, incubated, and then centrifuged per the manufacturer's instructions.

#### 2.4.1. Modified Protocol for Chondrogenic Differentiation mRNA Isolation

Briefly, the aqueous layer from the final TRIzol step was transferred into a HiBind RNA Mini Column and centrifuged. RNA Wash Buffer I was added to the column and recentrifuged. RNA Wash Buffer II was added and centrifuged; this was repeated twice. DEPC water was added to the column and centrifuged, and the mRNA was stored at −80°C until use.

#### 2.4.2. Osteogenic or Adipogenic Differentiation mRNA Isolation

The aqueous layer from the TRIzol reagent was supplemented with isopropanol, vortexed, incubated, and then centrifuged. The resultant pellet was washed with 75% ethanol and centrifuged. The mRNA pellet was resuspended with 20 *μ*L RNA of ultrapure water (VWR) and stored at −80°C until use.

#### 2.4.3. First-Strand Synthesis

First-strand cDNA was generated from the extracted mRNA using the High-Capacity cDNA kit (Thermo Fisher Scientific) following the manufacturer's protocol. cDNA samples were stored at −20°C until use.

#### 2.4.4. Real-Time Quantitative PCR (qPCR) Analysis

For osteogenesis, the expression levels of *Osterix* (*Sp7*) and *Runx2* were quantified. For adipogenesis, *adiponectin* was measured. For chondrogenesis, *Sox9* and *Col2a* were examined. Ribosomal *18S* was employed as an internal control/housekeeping gene (all Taqman assayed from Thermo Fisher Scientific).

MicroAmp Optical Reaction Plates (Thermo Fisher Scientific) were employed as the reaction vessel. Master mix was made up for each of the probes/markers that contained 0.5 *μ*L of the specific probe, 5 *μ*L of TaqMan Universal PCR Master Mix No AmpErase (Applied Biosystems), and 3.5 *μ*L of ultrapure water. In each well, a final volume of 9 *μ*L of master mix was mixed with 1 *μ*L of the cDNA. Three replicates were run per sample.

Cycle threshold (CT) value (of the triplicates) for each marker obtained from qPCR data was calculated against the CT values from the 18S housekeeping gene.

### 2.5. Flow Cytometry

At the point where HEF-derived cells were placed under differentiation conditions, an aliquot of the cells was also examined for cell surface marker expression using flow cytometry. The cells were isolated using trypsin and then stained with CD271, CD105, CD90, CD73, and CD44 (all from BD Biosciences, Franklin Lakes, New Jersey).

### 2.6. Collagen Contraction Assay

Purified bovine type I collagen (3 mg/mL, Advanced BioMatrix) was mixed with HEF cells to obtain a final cell concentration of 1 × 10^5^ cells/mL. The collagen/cell mixture was polymerised using 1 M NaOH. The resulting gel was displaced from the culture well thereby allowing contraction.

### 2.7. Histology

To complement the qPCR analysis, histological analysis was performed at the completion of differentiation. Briefly, cells were fixed with 10% neutral buffered formalin (NBF) for one hour at room temperature and then stained for a specific histological reagent for each lineage examined. Osteogenic cultures were stained with Alizarin Red to detect calcium (Sigma-Aldrich). Adipogenic cultures were stained with Oil Red O solution to detect lipids (Sigma-Aldrich). The chondrogenic cultures were stained with Safranin O to detect glycosaminoglycans (Sigma-Aldrich).

### 2.8. Lineage Tracking

All mice were handled in accordance with the recommendations in the Canadian Council on Animal Care Guidelines, and the animal protocol was approved by the University of Calgary Animal Care Committee. The *Prx1*/*Prrx1-cre/ERT2,-EGFP^1Smkm^*/J mouse line used in this study was kindly provided by Dr. Shunichi Murakami (Case Western Reserve, Cleveland, OH). *Gt(ROSA)26Sor^tm14(CAG-tdTomato)Hze^*/J mice were purchased from The Jackson Laboratory. To generate the transgenic mice used, the following breeding scheme was employed: *Prx1-cre/ERT2,-EGFP^1Smkm^*/J^+/+^ mice were bred to *Gt(ROSA)26Sor^tm14(CAG-tdTomato)Hze^*/J^+/+^ mice to generate *Prx1-cre/ERT2,-EGFP^1Smkm^*/J^+/−^;*Gt(ROSA)26Sor^tm14(CAG-tdTomato)Hze^*/J^+/−^. The active isomer of tamoxifen ((Z)-4-hydroxytamoxifen, Sigma-Aldrich) was dissolved in filter-sterilized sunflower oil to make solutions with a final concentration of 10 mg/mL. Mice were injected with approximately 100 *μ*L of tamoxifen solution (100 mg/kg) once a day over 5 days, followed by a 1-week waiting period to allow for recombination.

Intact mouse spines were dissected and fixed in neutral buffered formalin (Sigma-Aldrich) for 5 days, then decalcified in Cal-Ex (Fisher Scientific) for 10 days. After the 10-day decalcification procedure, samples underwent tissue processing for paraffin sectioning. Serial sagittal sections (10 *μ*m) were costained with Safranin O and Fast Green. To visualize Safranin O staining or the endogenous fluorescence (EGFP or TdTomato), an Axio Scan.Z1 Slide Scanner microscope (Carl Zeiss) outfitted with a Plan-Apochromat objective (10x/0.8 M27) was used to image slides. The following filters were applied: DAPI (353 nm/465 nm), EGFP (493 nm/517 nm), and DsRed (563 nm/581 nm).

## 3. Results

### 3.1. Differentiation Potential

Cells isolated from HEF were evaluated for their multipotent differentiation capacity. Molecular (qPCR) and histological outcome measures were employed. Therefore, for a positive differentiation result into a specific lineage, a positive result for both qPCR and histology outcomes had to be obtained for a given cell line.

**Figure 1:**
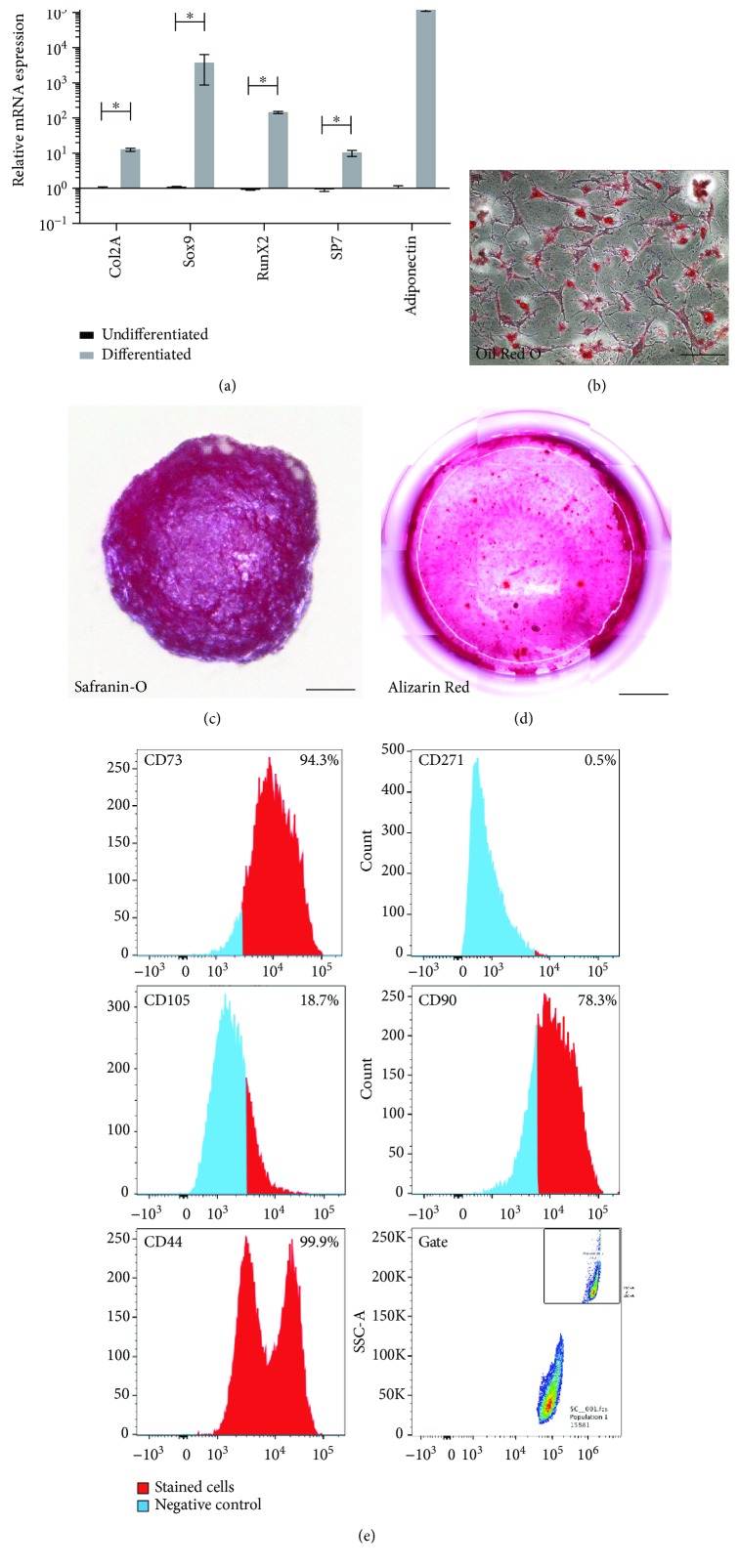
Characterization of MSCs from HEF. qPCR analysis demonstrated induction of *Col2a*, *Sox9*, *Runx2*, *Sp7*, and *adiponectin* after differentiation compared to untreated controls (a). Error bars represent ±SD (^∗^*p* < 0.05). Histological staining for Oil Red O (b), Safranin O (c), and Alizarin Red (d) was positive. Flow cytometry analysis demonstrated the HEF cells were positive for CD105, CD90, CD73, and CD44 (e). Scale bars represent 30 *μ*m (b), 100 *μ*m (c), and 2000 *μ*m (d).

**Figure 2:**
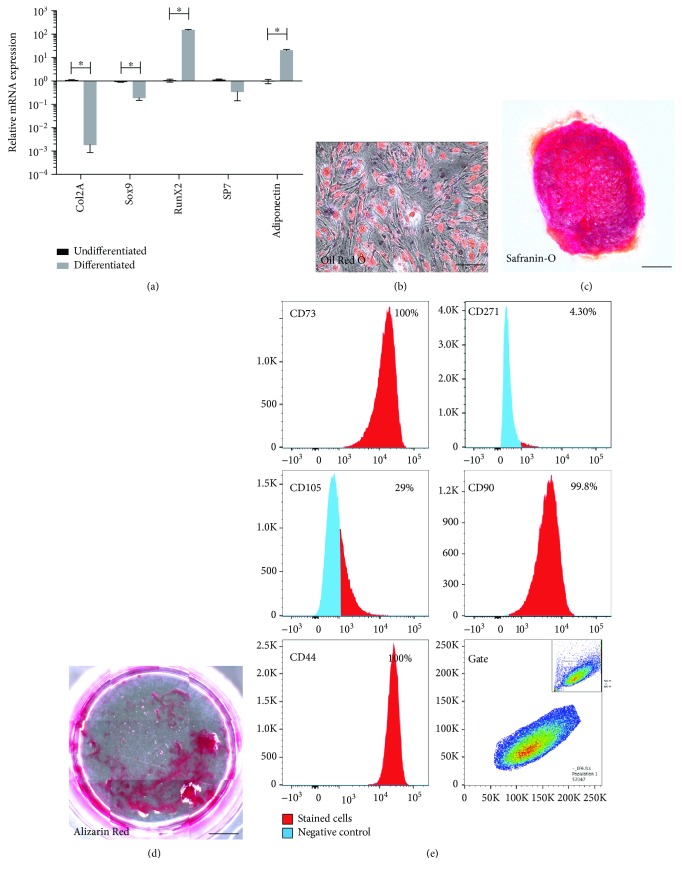
Characterization of bipotent progenitors from HEF. qPCR analysis demonstrated induction of *Runx2*, *Sp7*, and *adiponectin* after differentiation compared to untreated controls, while *Col2a* and *Sox9* were not increased (a). Error bars represent ±SD (^∗^*p* < 0.05). Histological staining for Oil Red O (b), Safranin O (c), and Alizarin Red (d) was positive. Flow cytometry analysis demonstrated the HEF cells were positive for CD105, CD90, CD73, and CD44 (e). Scale bars represent 30 *μ*m (b), 100 *μ*m (c), and 2000 *μ*m (d).

**Figure 3:**
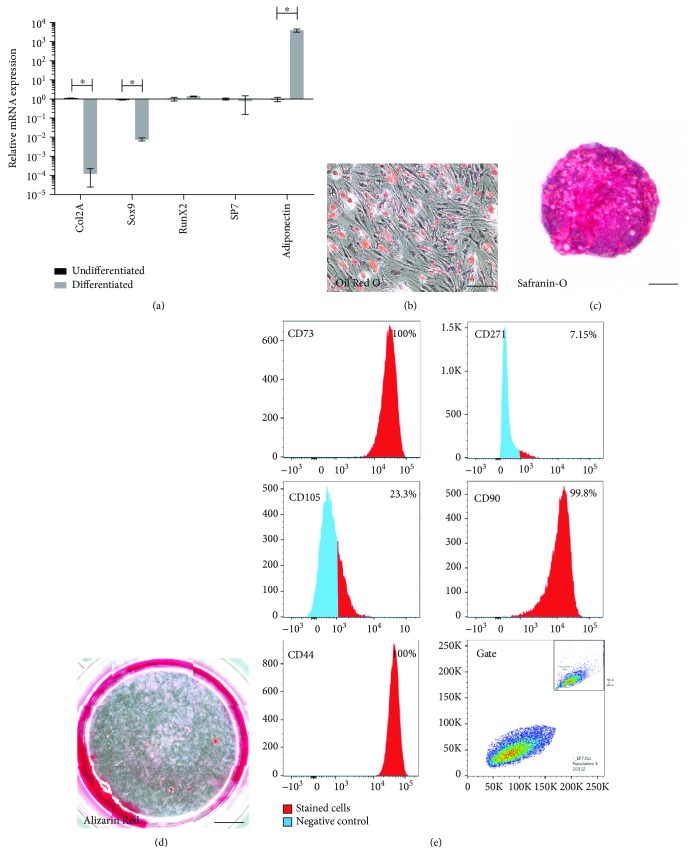
Characterization of unipotent progenitors from HEF. qPCR analysis demonstrated the induction of *adiponectin* after differentiation compared to untreated controls, while *Col2a*, *Sox9*, *Runx2*, and *Sp7* were absent (a). Error bars represent ±SD (^∗^*p* < 0.05). Histological staining for Oil Red (b) was positive while Safranin O (c) staining was weak and Alizarin Red (d) staining was absent. Flow cytometry analysis demonstrated the HEF cells were positive for CD105, CD90, CD73, and CD44 (e). Scale bars represent 30 *μ*m (b), 100 *μ*m (c), and 2000 *μ*m (d).

Three cell populations with distinct potencies within HEF were identified: cells with (1) trilineage (MSC), (2) bilineage, and (3) unilineage potential. A summary of all the population potentials are described in Table 1. The *in vitro* data presented in Figures 1–3 are representative results from one example of each type of stem/progenitor population.

To determine if patient age was associated with cell potency/potential, a correlation analysis was undertaken. No correlation between HEF potency and patient age was observed (*R* = −0.106, *p* = 0.590).

### 3.2. Mesenchymal Stem Cells

In 2 out of 10 patients, cells with multilineage (bone, cartilage, and fat) differentiation potential meeting the phenotypic definition of MSCs were isolated and characterized. Based on qPCR analysis (Figure 1), these cells exhibited an induction of mRNA expression for chondrogenic, osteogenic, and adipogenic markers after differentiation. Histological staining confirmed the molecular analysis of differentiation. The cells demonstrated positive staining for Safranin O (glycosaminoglycans), Alizarin Red (calcium), and Oil Red O (lipid) (Figure 1).

Cell surface proteins routinely used to identify MSCs were further examined using flow cytometry (minimum criteria by the International Society for Cellular Therapy (ISCT)) [40]. Flow cytometry data demonstrated that nearly all cells expressed CD73, CD90, and CD44, while CD105 was only expressed by a proportion of the population and CD271 expression was absent (Figure 1). Therefore, in accordance with the minimum criteria set by ISCT [40], cells derived from 2 out of 10 patients met the criteria to be defined as MSCs.

### 3.3. Bipotent Progenitors

In 6 out of 10 patients, cells with partial multilineage differentiation potential were isolated and characterized. These cell populations demonstrated a limited ability to differentiate compared to MSCs, yet they did demonstrate bilineage capacity. mRNA extracted from differentiated cells demonstrated an upregulation of osteogenic and adipogenic markers compared to undifferentiated cells isolated from the same patient; however, both *Col2a* and *Sox9* (chondrogenic markers) were downregulated/not expressed (Figure 2). Histological analysis demonstrated that these cells stained positive for Alizarin Red and Oil Red O confirming the qPCR results. However, cell pellets also stained positive for Safranin O, signifying the presence of glycosaminoglycans in the absence of chondrogenic markers (*Col2a* and *Sox9*) (Figure 2). The cell surface marker expression profile of these bipotent progenitor cells was similar to that of MSCs. Bipotent progenitor cells were positive for CD73, CD90, and CD44, while approx. 30% of the population was positive for CD105 and CD271 expression was absent (Figure 2).

### 3.4. Unipotent Potential

In 2 out of 10 patients, cells with limited multilineage differentiation potential were isolated and characterized. These cells lacked the ability to differentiate into chondrocytes or osteoblasts but retained the ability to differentiate into adipocytes. mRNA extracted from differentiated cells demonstrated an upregulation of adipogenic markers compared to undifferentiated cells isolated from the same patient; however, both chondrogenic and osteogenic markers were absent (Figure 3). Histological analysis demonstrated that these cells stained positive for Oil Red O (lipid) confirming the qPCR results. However, cell pellets also stained weakly positive for Safranin O, signifying the presence of glycosaminoglycans in the absence of chondrogenic markers (Figure 3). Alizarin Red staining was negative signifying the absence of calcium deposition (Figure 3).

The cell surface marker expression profile of these unipotent progenitor cells are similar to that of full MSCs and bipotent progenitors. Unipotent progenitor cells were positive for CD73, CD90, and CD44, while approx. 25% of the population was positive for CD105 and CD271 expression was absent (Figure 3).

### 3.5. Fibroblastic Activity

**Figure 4:**
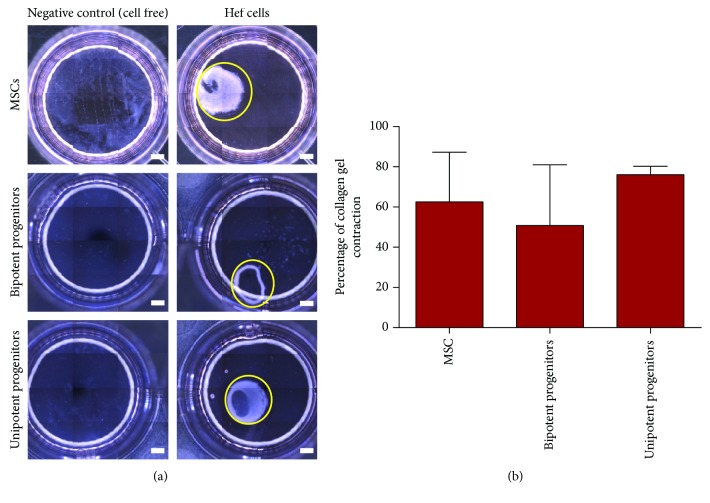
Collagen contraction assay. Representative images demonstrating contractile activity of HEF cell populations. The area of gel contraction at 24 hr postcell seeding is presented compared to negative (cell-free) control (a). There was no association between cell potency and collagen contraction ability (b). Scale bars represent 1000 *μ*m.

To determine if these cell populations from HEF demonstrate fibroblastic-like activity, collagen gel contraction assays were performed. All cell populations tested, regardless of potency, demonstrated a capacity for collagen gel contraction (Figure 4).

### 3.6. *In Vivo* Lineage Tracking

**Figure 5:**
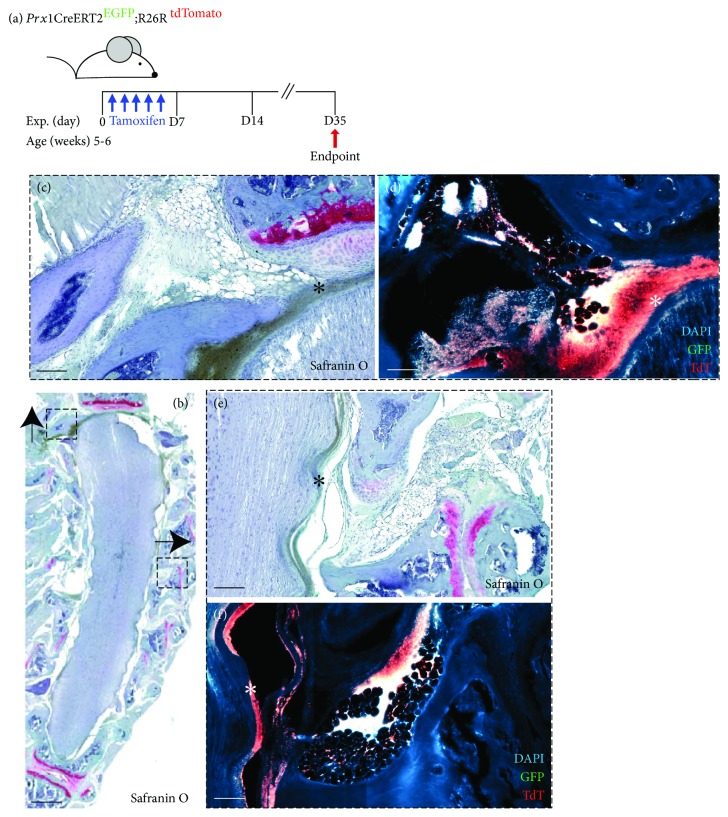
*Prx1* lineage reporter mouse. Diagram representing tamoxifen injection schedule and experimental endpoint (a). Representative histology and fluorescent reporter imaging (b-f) indicating that epidural fat contains *Prx1*+ cells (EGFP/green) and their lineage-traced progeny (TdTomato (TdT/red) can be identified in the dura (asterisk). Scale bars represent 50 *μ*m (c, d, e, and f) and 500 *μ*m (b).

To examine epidural fat MSCs *in vivo*, a Prx1 (adipose MSC marker) lineage reporter mouse was employed [40, 41]. Cells actively expressing *Prx1* co-express GFP, while cells expressing *Prx1* at the time of tamoxifen injections are also permanently labelled with tdTomato. *Prx1+* MSCs were found within the epidural fat tissue adjacent to the spinal cord (Figure 5). Furthermore, the progeny of *Prx1*+ cells (GFP-, tdTomato+) were found to be heavily enriched in the dura (Figure 5), specifically in areas where the epidural fat made connection to the dura.

## 4. Discussion

The key finding of the current study is that cells isolated from HEF are metabolically active and have the ability to differentiate into osteo-, chondro-, and adipogenic lineages.

In keeping with ISCT guidelines, cells isolated from 2/10 patients met the minimum criteria required for definition as an MSC by demonstrating both self-renewal capacity and differentiation ability [42]. The remainder of patient cell lines demonstrated either bi- (6/10) or single lineage (2/10) potential. Cell surface marker analysis demonstrated the presence of MSC markers (CD90+, CD73+, CD44+, and CD105+) across all patient-derived cell lines with no association between marker expression and differentiation potential observed. Additionally, the cell contractility assay was also positive across all samples indicating either the presence of a fibroblast population in HEF or the presence of these HEF MSCs/progenitors also demonstrating an activated fibroblast phenotype.

To further confirm the presence of MSCs within epidural fat *in vivo*, *Prx1* reporter mice were employed and they demonstrated positive staining of putative MSCs within the epidural fat. Interestingly, it was observed that in areas where the epidural fat came into contact with the dura of the spinal cord, the dura was enriched for the progeny of *Prx1+* cells. The cellular origin of the dura and what cell populations maintain this tissue through adulthood are still unclear. Therefore, additional study will be required to fully characterize the contribution of *Prx1* + cells to the dura and to validate that these cells are derived from the epidural fat.

It remains unclear as to why some patient samples contained cells that were solely committed to the adipose lineage compared to others which remained as MSCs; however, it could be postulated that the effect could be due to age and/or severity of disease. A larger study with normal control samples would be required to test this hypothesis.

In the current study, we have presented data which suggests a novel biological activity of HEF. At this point, it remains unknown if/how these cells interact with their local environment; however, it is not unreasonable to consider that HEF serves as a reservoir of cells for local tissue maintenance/regeneration as well as serving as a local immunomodulatory and cell signalling tissue, in keeping with the role of fat in other anatomical sites. Adipose tissue has been studied in great detail, and it is firmly established as a tissue with potent biological activity and capacity serving a number of local and systemic functions, not to mention its potential as a regenerative tissue source [5, 6, 8, 9, 19, 28, 29, 33, 38, 43, 44]. Scientifically and intraoperatively, epidural fat still receives little or no attention and is considered largely to be irrelevant clinically. The outcome of this *ex vivo* study is to change this perception and expand upon the role proposed by Beaujeux, as well as to highlight that further study be considered to examine the homeostatic and regenerative potential of HEF.

## Figures and Tables

**Table 1:** A summary for MSCs, bipotent cells, and unipotent cells that have been identified from 10 patients. Based on qPCR, histology, and flow cytometry data.

Age	Sex	Cell potency	Cell surface marker expression (percent of positive cells)					Chondrogenic differentiation capacity	Osteogenic differentiation capacity	Adipogenic differentiation capacity	Passage number of cells at time of testing	~Population doublings of cells at time of testing
			CD271	CD105	CD90	CD73	CD44					
55	M	MSCs (2/10 patients)	5%	29%	85%	100%	100%	Positive	Positive	Positive	4	6.78
70	M		<1%	19%	79%	95%	100%	Positive	Positive	Positive	4	7.26

66	F	Bipotent progenitors (6/10 patients)	<1%	73%	97%	100%	100%	Negative	Positive	Positive	4	5.38
64	M		<1%	95%	99%	100%	100%	Negative	Positive	Positive	4	7.35
47	M		5%	24%	75%	74%	75%	Negative	Positive	Positive	4	7.55
60	F		5%	55%	99%	100%	100%	Negative	Positive	Positive	4	8.36
24	F		4%	30%	99%	100%	100%	Negative	Positive	Positive	4	8.11
80	M		15%	55%	99%	100%	100%	Positive	Positive	Negative	4	7.08

67	F	Unipotent progenitors (2/10 patients)	5%	29%	74%	75%	75%	Negative	Negative	Positive	4	7.75
73	F		7%	23%	100%	100%	100%	Negative	Negative	Positive	4	7.26

## Data Availability

Raw data and the necessary details can be provided by the corresponding author under reasonable request.
